# Measuring Turnover of SIV DNA in Resting CD4+ T Cells Using Pyrosequencing: Implications for the Timing of HIV Eradication Therapies

**DOI:** 10.1371/journal.pone.0093330

**Published:** 2014-04-07

**Authors:** Jeanette C. Reece, Alexey Martyushev, Janka Petravic, Andrew Grimm, Shayarana Gooneratne, Thakshila Amaresena, Robert De Rose, Liyen Loh, Miles P. Davenport, Stephen J. Kent

**Affiliations:** 1 Department of Microbiology and Immunology, University of Melbourne, Victoria, Australia; 2 Centre for Vascular Research, University of New South Wales, Kensington, NSW, Australia; University of Pittsburgh, United States of America

## Abstract

Resting CD4+ T cells are a reservoir of latent HIV-1. Understanding the turnover of HIV DNA in these cells has implications for the development of eradication strategies. Most studies of viral latency focus on viral persistence under antiretroviral therapy (ART). We studied the turnover of SIV DNA resting CD4+ T cells during active infection in a cohort of 20 SIV-infected pigtail macaques. We compared SIV sequences at two Mane-A1*084:01-restricted CTL epitopes using serial plasma RNA and resting CD4+ T cell DNA samples by pyrosequencing, and used a mathematical modeling approach to estimate SIV DNA turnover. We found SIV DNA turnover in resting CD4+ T cells was slow in animals with low chronic viral loads, consistent with the long persistence of latency seen under ART. However, in animals with high levels of chronic viral replication, turnover was high. SIV DNA half-life within resting CD4 cells correleated with viral load (p = 0.0052) at the Gag KP9 CTL epitope. At a second CTL epitope in Tat (KVA10) there was a trend towards an association of SIV DNA half-life in resting CD4 cells and viral load (p = 0.0971). Further, we found that the turnover of resting CD4+ T cell SIV DNA was higher for escape during early infection than for escape later in infection (p = 0.0084). Our results suggest viral DNA within resting CD4 T cells is more labile and may be more susceptible to reactivation/eradication treatments when there are higher levels of virus replication and during early/acute infection.

## Introduction

The clinical outcome for HIV-infected individuals has improved dramatically since the development of potent combination antiretroviral therapies (cART) [1], [2]. Upon the cessation of treatment, however, viral replication is quickly re-established due to the presence of latent reservoirs, such as the resting CD4+ T cell pool [3]–[6].

Several eradication studies aimed at purging HIV-1 from the latent reservoir are currently in progress [7]–[9]. Preliminary results of clinical studies of purging using current drugs suggests that these may have only a small impact on the total latent reservoir [10]–[14]. It is likely there will need to be a better use of current agents, perhaps in combination with newer agents, to have a clinically useful benefit in reducing the latent reservoir.

Understanding the stability and persistence of the latent reservoir has important implications for optimising the effectiveness of these strategies [15]. The majority of studies of HIV DNA turnover and latency have been performed under ART, where a very slow turnover of HIV DNA is observed [5], [16]–[23]. However, little is known about the turnover of HIV DNA during active infection, and whether this may be a better time for interventions to reduce latency. SIV infection of macques provides a model to study the dynamics of latent HIV infection where the timing and strain of the infection is known.

Resting CD4 T cells in blood are probably a singificant reservoir of latent HIV and SIV infection and readily sampled over time. Other blood cells, including antigen-presenting cells, as well as cells in other tissues are also likely to be singificant reservoirs of latent HIV and SIV although are less well studied. We previously developed a novel approach to measuring SIV DNA turnover in resting CD4+ T cells during active SIV infection of macaques, by studying the rate of change of viral immune escape mutants in serial plasma RNA and in resting CD4+ T cell SIV DNA samples, an approach that we termed the ‘escape clock’ for measuring latency turnover [24]. That approach utilized a quasispecies-specific qRT-PCR [25] that was able to measure the frequency of wild type (WT) and escape mutant virus (EM) at a Mane-A1*084:01-restricted epitope in Gag that we termed KP9. While the rate of escape from the wildtype KP9 sequence to the escape mutant (K165R-EM) sequence was rapid in plasma, the time taken for the K165R-EM mutant to accumulate in the DNA of resting CD4+ T cells was variable. A delay in the appearance of the mutant in the resting CD4 T cell DNA would suggest a slowly turning over reservoir. Using a mathematical modelling approach, we showed that the rate of turnover of SIV DNA in resting CD4+ T cells was highly dependent on the viral load of the infected macaques, with extremely high rates of SIV DNA turnover seen in animals with high chronic viral loads [15], [24].

The observation of high SIV DNA turnover during active infection has important implications for strategies aimed at ‘purging’ the SIV reservoir. For example, one prediction from the “escape clock” result is that the higher levels of viral replication during early SIV or HIV-1 infection would lead to higher levels of turnover of the latent reservoir during early infection. This hypothesis is relevant to determining the optimal time to begin treatment with both purging drugs and cART, as recent studies have reported lower frequencies of latently infected cells as a consequence of very early cART treatment [26]–[30].

One limitation of the previous approach was the reliance on a quasispecies-specific qRT-PCR, which is only useful in the context of a specific KP9 escape mutation. Here we attempted to validate of the “KP9 escape clock” model of SIV DNA half-life in resting CD4 T cells using pyrosequencing for both the KP9 epitope, as well as another Mane-A1*084:01-restricted epitope in Tat, which we termed KVA10. Overall, our pyrosequencing results confirmed our earlier conclusions about the relationship between chronic viral load and SIV DNA stability, and showed that pyrosequencing is a useful approach for understanding and quantifying quasi-species turnover. Further, we analyzed CD4+ T cell SIV DNA turnover early during infection compared to during chronic infection, and found higher levels of turnover of SIV DNA in resting CD4 T cells during early SIV infection.

## Results

### KP9 escape using pyrosequencing compared to qRT-PCR

**Figure 1:**
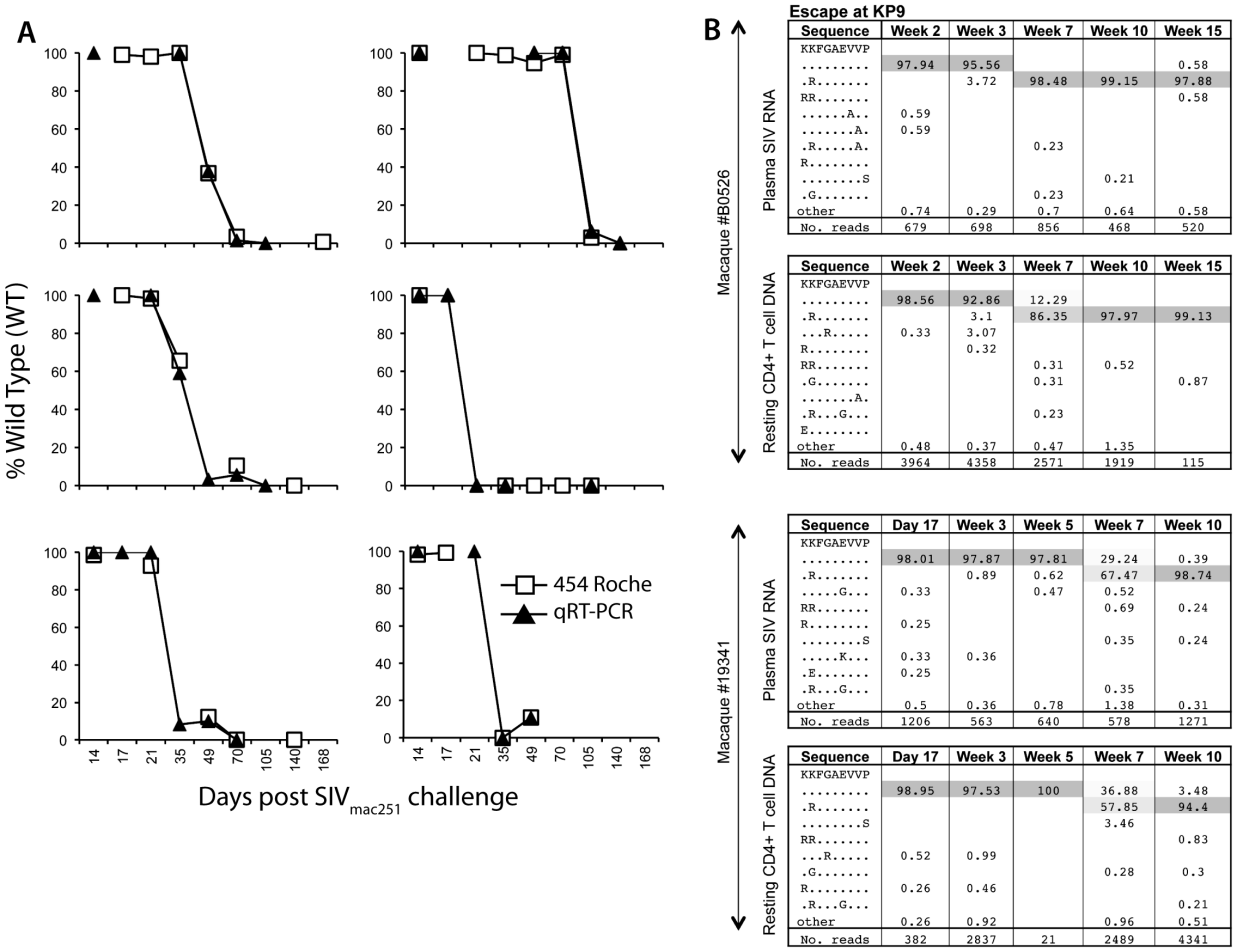
Analysis of escape at the KP9 Gag CTL epitope by pyrosequencing. A. Estimation of K165R KP9 escape in serial resting CD4+ T cell SIV DNA samples using pyrosequencing compared to KP9-specific qRT-PCR. Six representative macaque examples comparing CTL escape at KP9 from serial resting CD4+ T cell SIV DNA samples after infection with SIV_mac251_ determined using the KP9-specific qRT-PCR compared to pyrosequencing. B. KP9 escape in plasma SIV RNA and resting CD4+ T cell SIV DNA for 2 representative macaques using Roche 454 sequencing. Examples of KP9 CTL escape in plasma SIV RNA and resting CD4+ T cell SIV DNA 2 animals using pyrosequencing. The CTL amino acid sequence is shown in the first column, with the % of sequence in the subsequent columns and the time point post SIV challenge at the top of the column. The mutation identified is shown at each time point with the total reads shown in the bottom row. Common variants at each time point are shaded with rarer variants accounting for the remaining sequences.

We first analysed the evolution of immune escape at the KP9 epitope in resting CD4 T cells comparing the pyrosequencing data to the qRT-PCR data. We found that the proportion of KP9 WT virus in resting CD4+ T cell SIV DNA from animals obtained using nested pyrosequencing was very similar to the proportion of KP9 WT virus estimated using the nested KP9-specific qRT-PCR (Figure 1A).

KP9 escape in plasma SIV RNA was then directly compared with KP9 escape in SIV DNA from resting CD4+ T cells in SIV-infected pigtail macaques by pyrosequencing. Pyrosequencing enabled the timing and nature of escape across the KP9 epitope in both plasma SIV RNA and resting CD4+ T cell SIV DNA to be determined (illustrated in two animals in Figure 1B).

### KP9 escape by pyrosequencing and the “escape clock”

**Figure 2:**
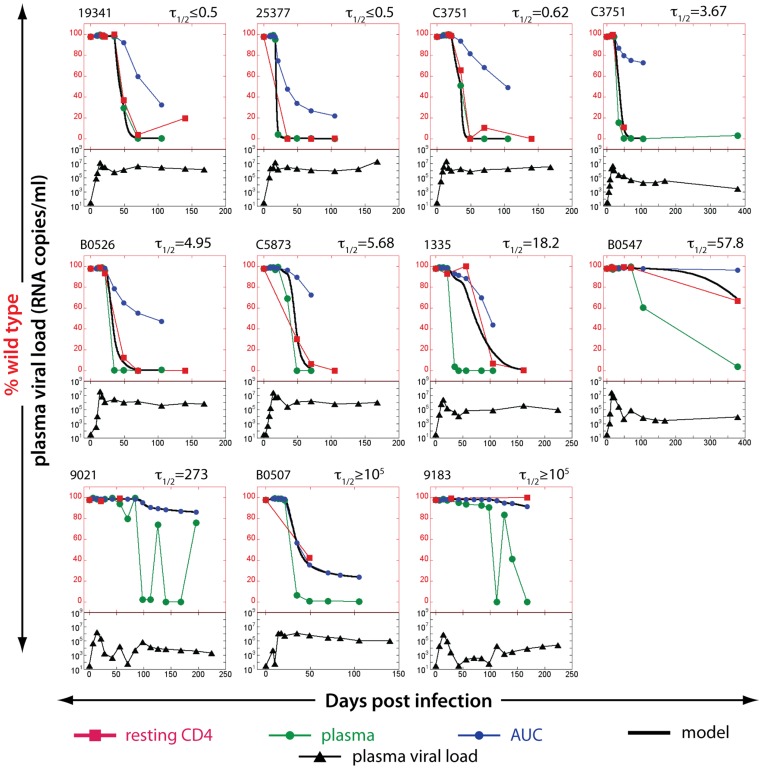
Estimating the half-life of SIV DNA in resting CD4+ T cells studying KP9 escape using pyrosequencing data. The proportion of WT virus in plasma (green circles), the fraction of WT virus estimated from area under the curve (AUC) of viral load (blue circles) and the experimentally observed fraction of WT virus SIV DNA in resting CD4+ T cells (red squares) for each animal in the top of each figure. The black line represents the line of best-fit SIV DNA half-life to the observed fraction of WT virus in resting CD4+ T cells for each animal. Animals are arranged in the order of increasing estimated lifespan. Total plasma viral loads (log_10_ scale, from 10–10^9^) are illustrated in the bottom part of each figure (black triangles). The lack of data at crucial time points made it impossible to estimate the life spans of resting infected cells in 9 out of 20 animals.

Our analysis of plasma viral sequences at the KP9 epitope showed rapid replacement of the WT virus with EM virus. However, the rate of loss in WT virus in resting CD4+ T cells was variable, reflecting the variable half-life of SIV DNA in these cells. The ratios of WT:EM virus detected using the KP9-specific qRT-PCR assay on plasma SIV RNA and resting CD4+ T cell SIV DNA allows us to estimate the turnover of SIV-DNA using mathematical modeling (the “escape clock” model, Eq. 2) [24]. Serial measurements of the frequency of different viral variants at the KP9 epitope were obtained by pyrosequencing in samples of 11 out of the 20 macaques [24]. The remaining 9 animals had insufficient longitudinal samples to estimate the turnover of SIV DNA. We noted that 2 animals with delayed escape kinetics in plasma RNA at the KP9 epitope (#9021 and #9183) had fluctuating levels of escape once escape began (Fig. 2, lower panels). We speculate that slower and weaker generation of CTL pressure for escape may result in fluctuating level of escape in these 2 animals.

Using pyrosequencing data, we estimated the turnover of SIV DNA in infected animals. This approach relies on the fact that the WT virus is present in the replicating pool for a limited period in early infection, and then largely disappears from the replicating virus in late infection. Thus, latently infected cells carrying WT virus were laid down early in infection, and measuring the persistence of WT DNA in resting CD4+ T cells tells us about the duration of SIV latency. Consistent with our previous results, we found that in animals with high viral loads, KP9 escape in resting CD4+ T cells closely followed KP9 escape in plasma SIV RNA, suggesting a high rate of turnover of SIV DNA in these cells (Figure 2, top row). Using the “escape clock” to estimate the SIV DNA turnover rate in resting CD4+ T cells, the half-life of SIV DNA in these animals was estimated to be extremely short (in the order of a few days). In contrast, in animals with prolonged low levels of viral replication the KP9 epitope sequences from resting CD4+ T cells remained close to 100% WT, despite the dominance of EM in the plasma (Figure 2, bottom row). The half-life of SIV DNA in these resting CD4+ T cells was estimated to be extremely long, suggesting that SIV DNA in these cells is very long-lived (in the order of years), consistent with previous studies of HIV DNA persistence under drug therapy [20]. These results are consistent with our previous “KP9 escape clock” hypothesis using the KP9-specific qRT-PCR [24].

**Figure 3:**
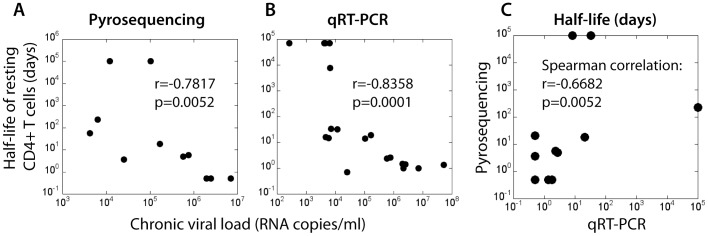
Half-life of resting CD4+ T cell SIV DNA decreases with increasing chronic plasma viral load. The chronic plasma viral load (geometric mean viral load from day 100 post-infection) is significantly negatively correlated with the estimated half-life of SIV DNA for each animal. Half-life estimated using (A) pyrosequencing data (Spearman correlation, r = −0.7817, p = 0.0052) and (B) KP9-specific q-RT-PCR data (Spearman correlation, r = −0.8358, p<0.0001). C. Half-life estimations' (days) using pyrosequencing compared to the KP9-specific qRT-PCR are significantly correlated (Spearman correlation, r = 0.6682, p = 0.0297).

To investigate this observation further, we looked for a correlation between chronic viral load and estimated resting CD4+ T cell SIV DNA half-life using pyrosequencing data (Figure 3A). In agreement with previous findings observed using the KP9-specific qRT-PCR [24] (shown in Figure 3B), a significant association between the average viral load in chronic infection and the estimated rate of SIV DNA turnover in resting CD4 T cells was observed. When we compared the half-lives of SIV DNA in resting CD4 T cells across the 2 methodologies (pyrosequencing and qRT-PCR) we found a significant correlation (r = 0.67, p = 0.03, Figure 3C).

### Measuring SIV DNA turnover at the Tat KVA10 epitope

The results above use two different methods of quantitation to study escape at the same epitope. To determine if these results could be replicated by studying escape at another SIV CTL epitope, we performed pyrosequencing across the KVA10 Tat CTL epitope using serial resting CD4+ T cell SIV DNA and plasma SIV RNA samples from the same animals. The KVA10 epitope usually escapes early, similar to KP9 escape [31], [32]. However, whereas escape at the KP9 epitope usually results in the same K165R mutation in most animals, escape at KVA10 is more diverse and polymorphic between animals [31], [32]. As a result, escape can only be measured across multiple animals by sequencing methods rather than a qRT-PCR. To enable detection of KVA10 escape in resting CD4+ T cell SIV DNA using pyrosequencing, a first round Tat-specific PCR was employed followed by second round KVA10-specific PCRs using unique combinations of MID-tagged oligonucleotides.

**Figure 4:**
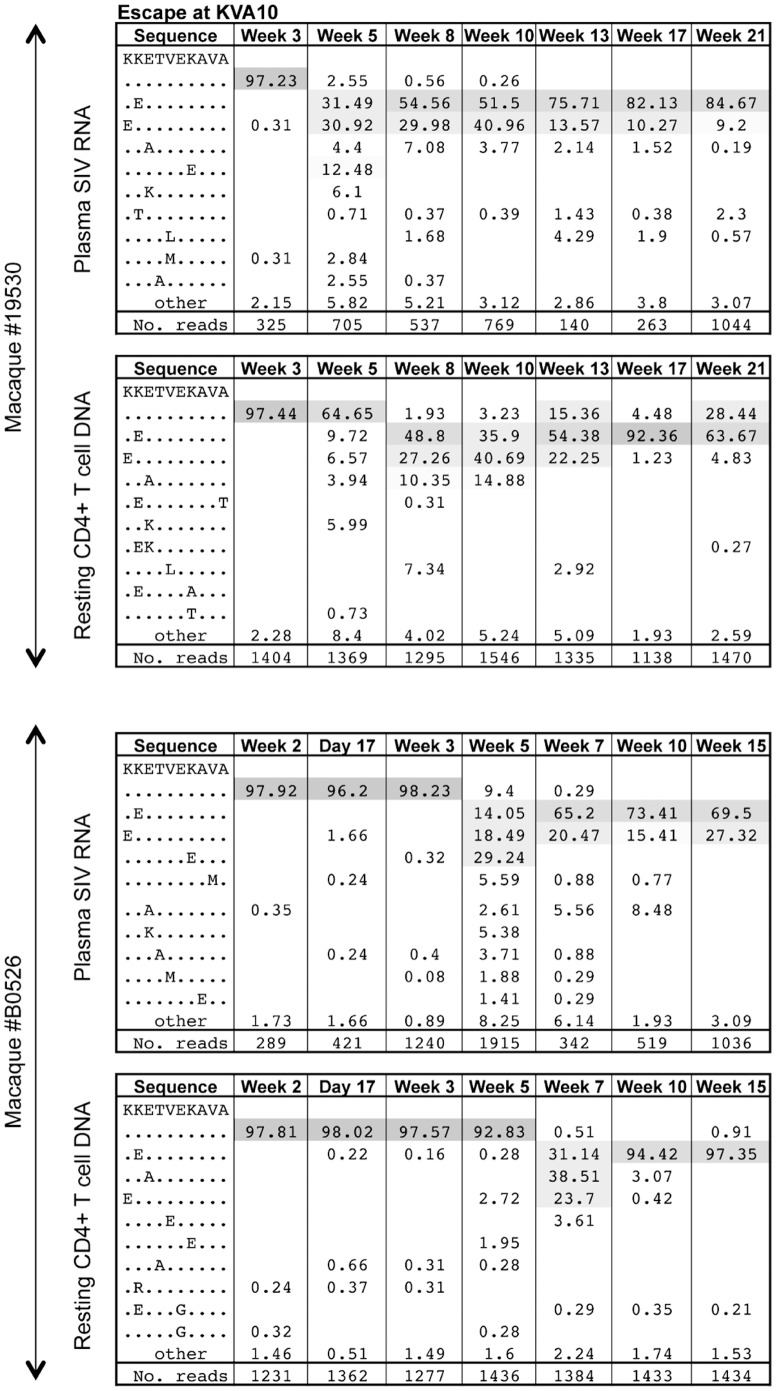
KVA10 escape for plasma SIV RNA and resting CD4+ T cell SIV DNA using pyrosequencing. Examples of KVA10 CTL escape in plasma SIV RNA and resting CD4+ T cell SIV DNA in 2 animals by pyrosequencing. The CTL amino acid sequence is shown in the first column, with the % of sequence in the subsequent columns and the time point post SIV challenge at the top of the column. The mutation identified is shown at each time point with the total reads shown in the bottom row. Common variants at each time point are shaded and rarer variants account for the remaining sequences.

KVA10 escape from serial plasma SIV RNA and resting CD4+ T cell SIV DNA samples following SIV_mac251_ infection of two representative animals measured using pyrosequencing is shown in Figure 4. This figure illustrates the polymorphic and diverse nature of KVA10 escape in macaques.

**Figure 5:**
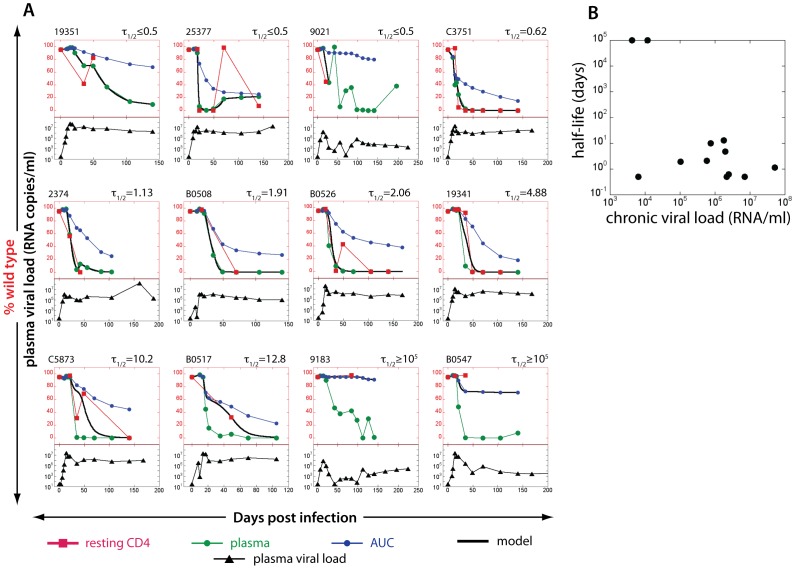
Estimating the half-life of SIV DNA in resting CD4+ T cells studying KVA10 escape using pyrosequencing data. A. The proportion of WT virus in plasma (green circles), the fraction of WT virus estimated from area under the curve (AUC) of viral load (blue circles) and the experimentally observed fraction of WT virus SIV DNA in resting CD4+ T cells (red squares) for each animal in the top of each figure. The black line represents the line of best-fit SIV DNA half-life to the observed fraction of WT virus in resting CD4+ T cells for each animal. Animals are arranged in the order of increasing estimated lifespan. Total plasma viral loads (log_10_ scale, from 10–10^7^) are illustrated in the bottom part of each figure (black triangles). 12 animals with sufficient data on RNA and DNA escape were available to study. B. Correlation between half-life of SIV DNA in resting CD4+ T cells with chronic plasma viral load for KVA10 epitope using pyrosequencing. Spearman correlation, r = −0.4138, p = 0.0971.

We were able to obtain multiple timepoints of plasma and resting CD4+ T cell sequences at the KVA10 epitope from 12 of the 20 animals to estimate the turnover of SIV DNA in (Figure 5). The other 8 animals had too few data points for this analysis. Consistent with our results using the KP9 epitope, we found that the rate of replacement of the WT KVA10 epitope was very high in resting CD4+ T cell SIV DNA in animals with high viral load (see examples in upper and middle rows of Figure 5A), suggesting a high turnover of resting CD4+ T cell SIV DNA. In contrast, replacement of WT KVA10 in resting CD4+ T cell SIV DNA in animals with low viral loads (such as #547 and #9175) is very much delayed compared to plasma SIV RNA (see examples in lower row of Figure 5A), suggesting the resting CD4+ T cell SIV DNA turnover in these animals is low.

Using the KVA10 escape data available, we investigated the correlation between resting CD4+ T cell SIV DNA half-life and chronic viral load (Figure 5B). There was a strong trend towards a correlation between the half-life of resting CD4 T cell SIV DNA and viral load using the KVA10 escape data using a two-tailed test (r = −0.4138, p = 0.0971).

### Turnover of SIV DNA in resting CD4 T cells during acute infection

The relationship between high turnover (short half-life) of SIV DNA in resting CD4 T cells and high chronic viral loads observed by pyrosequencing adds support to the suggestion that high levels of viral replication, and CD4+ T cell activation, may have a role in driving SIV DNA turnover in resting CD4+ T cells during active infection [24]. A prediction arising from this is that turnover of SIV DNA within resting CD4 T cells would be higher during early infection, when viral levels are typically high. We therefore aimed to assess the turnover of SIV DNA in resting CD4 T cells at different times post-infection. That is, we asked if escape occurs early during infection in the plasma, is the turnover of SIV DNA in resting CD4 T cells fast, and if escape occurs later in infection, is the turnover of SIV DNA in resting CD4 T cells slow. This analysis allows us to use the natural variability of time of escape at KP9 to assess the influence of timing of escape on the turnover of the SIV DNA reservoir.

**Figure 6:**
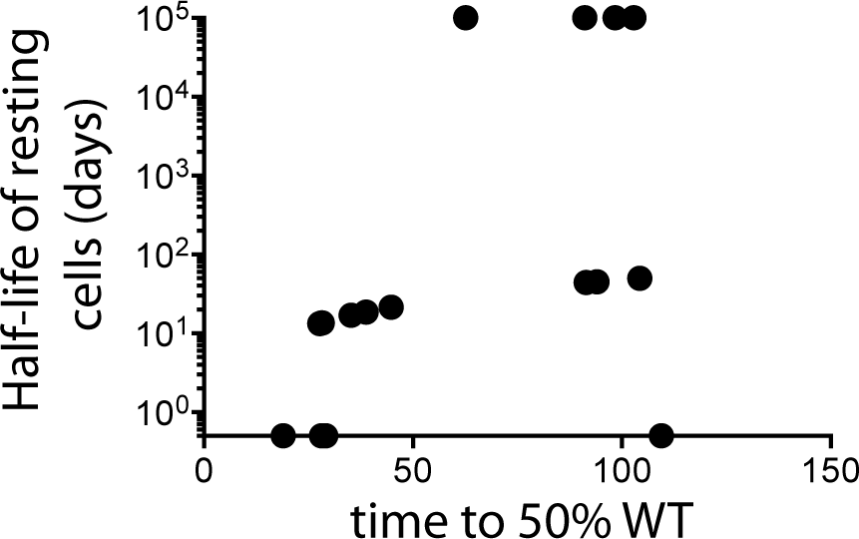
Turnover of SIV DNA in resting CD4+ T cells is higher during early infection. Relationship between timing of escape in plasma SIV RNA (time to reach 50% escape) at the KP9 epitope using the qRT-PCR and turnover of SIV DNA in resting CD4+ T cells. This analysis used data on 18 animals.

We assessed the relationship between time of escape and SIV DNA turnover using the escape rates at the KP9 Gag epitope using the qRT-PCR data, since we had robust data on 18 of the 20 animals. The other 2 animals had too few data points for this analysis. When we plotted turnover of SIV DNA in resting CD4+ T cells [half-life of resting cells (days)] against the imputated time at which there was 50% escape, we found higher rates of turnover of SIV DNA in resting CD4 T cells when escape occurred early (Fig. 6, p = 0.0084).

## Discussion

Our previous studies examining the dynamics of the latent reservoir in SIV-infected pigtail macaques not on cART using an allele-specific PCR for the common KP9 CTL epitope mutation K165R suggested that the turnover of the latent reservoir can be surprisingly fast in animals with high plasma viral loads [15], [24]. We now confirm these findings using a deep sequencing approach for both the KP9 epitope and another CTL epitope, KVA10, which escapes in a more variable manner and is not amenable to an allele-specific PCR approach. The turnover of SIV DNA in resting CD4+ T cells [reported as half-life of resting CD4+ T cells (days)] was similar to that previously obtained using the KP9-specific qRT-PCR. We conclude that both methodologies (allele-specific PCR and deep sequencing) yielded similar results and validate our findings on the effect of viral load on the turnover of total SIV DNA in resting CD4 T cells.

The latent HIV-1 DNA reservoir in resting CD4+ T cells is very long-lived at low viral loads (that is, during cART) [20], [33]–[35]. The persistence of SIV DNA in resting CD4 T cells in our study, however, was only seen in macaques with low chronic viral loads. Conversely, at high chronic viral loads, pyrosequencing confirmed the novel concept of high SIV DNA turnover during active infection, consistent with previous results. [24].

To further investigate whether the high turnover of SIV DNA in resting CD4+ T cells could be observed at another CTL epitope, we examined the rate of escape at the immunodominant SIV Tat KVA10 epitope using nested pyrosequencing and estimated the turnover of SIV using a modeling approach. The dynamics of escape in plasma virus and resting CD4+ T cell DNA at the KVA10 epitope showed a strong trend towards faster SIV DNA turnover in resting CD4+ T cells at high chronic viral load (p = 0.097, two tailed test). The KVA10 epitope escapes with a more variable pattern compared to the KP9 epitope and the limited number of animals for which longitudinal data were available for this analysis likely reduced our power to detect a significant association.

Given the association between high viral load and fast SIV DNA turnover, it seems likely that the latent viral reservoir may be more labile during acute infection. We explored this further by estimating the turnover of SIV DNA in resting CD4+ T cells during early untreated SIV infection compared to chronic infection. We found a significant association between the turnover of SIV DNA in resting CD4 T cells and the timing of escape – when escape occurred early in infection, there was a faster turnover of SIV DNA in resting CD4 T cells.

The rapid turnover of latently infected CD4+ T cells at high viral loads may be a direct result of increased immune activation during periods of uncontrolled viremia. Consequently, when virus replication is high, increased immune activation may also cause an increase in the activation of latently infected CD4+ T cells resulting in an increase in the turnover and subsequent loss of latently infected cells.

A limitation of our studies, however, is its inability to directly address the issue that the SIV DNA sequenced from resting CD4+ T cells may contain a mixture of both integrated SIV DNA and short-lived unintegrated SIV DNA. We estimated KP9 and KVA10 escape from total SIV DNA lysed directly from FACS sorted resting CD4+ T cells. Although the frequency of latent cells harbouring unintegrated SIV DNA during cART is estimated to be low [36]–[38], higher levels of linear unintegrated SIV DNA during untreated HIV-1 infection may be present [38]–[40]. Due to the low numbers of FACS sorted resting CD4+ T cells available from pigtail macaques, however, it was not possible to utilise methods to allow integrated SIV DNA to be discriminated from unintegrated SIV DNA [41], [42]. Future studies could focus on only integrated SIV DNA, for example using Alu-PCR methods, and use even more stringent methods to define resting CD4+ T cells.

Reservoirs of latent HIV other than in circulating resting CD4 T cells also exist, including in antigen-presenting cells and throughout multiple tissues. We have not measured the impact of viral load or early infection on turnover in these populations. This could be done in future studies by also FACS-sorting monocytes and other cell populations from both blood and, for example, serial lymph node biopsies or aspirates. One might expect that the high levels of generalized immune activation in untreated HIV and SIV infection would also lead to high turnover of reservoirs in immune cells other than resting CD4 T cells throughout the body, but this remains to be proven.

In summary, pyrosequencing can be employed to measure escape at different CTL epitopes in both plasma SIV RNA and SIV DNA in resting CD4+ T cells. Our results confirm a relationship between turnover of resting CD4+ T cell SIV DNA and chronic viral load in macaques not on antiretroviral treatment. Importantly, we also found a significantly higher turnover of latently infected cells during acute infection compared to during chronic infection. We suggest the testable hypothesis that treatment with drugs aimed at ‘purging’ the latent will be most effective when the turnover of resting CD4+ T cell SIV DNA is high and thus more susceptible to reactivation and elimination. This approach could alo be tested in humans with HIV-1 infection. This approach is expected to be more effective at purging the virus than treatment with purging agentswhen the reservoir is more stable, as occurs under long term cART. Moreover, we suggest that early treatment with both cART and reactivating purging drugs during acute infection may have the potential to decrease the size of the latent reservoir even further.

## Materials and Methods

### Animals

Twenty juvenile Mane-A1*084:01 positive pigtail macaques weighing 3–6 kg were infected with SIV_mac251_ (100% wild type at KP9) as previously described [24]. The Mane-A1*084:01 allele is the restriction factor for three SIV epitopes, the KP9 CTL epitope in Gag and the KVA10 and KSA10 epitopes in Tat [31], [32]. These animals were from a number of previous studies and represent all Mane-A1*084:01 available to study. Briefly, 5 macaques were control macaques [43], two macaques received influenza viruses expressing only the KP9 CTL epitope [44] and 5 macaques received influenza viruses expressing KP9, KSA10 and KVA10 CTL epitopes [31]. Eight pigtail macaques were enrolled in a therapeutic peptide-based vaccine trial [45] and received ART (tenofovir and emtricitibine) from week 3 to week 10 post SIV infection. Either no treatment (controls) or a peptide immunotherapy treatment (overlapping 15 mer Gag peptides only or peptides from all 9 SIV proteins) was given at weeks 4, 6, 8 and 10 post SIV infection.

### Ethics statement

Experiments on pigtail macaques (Macaca nemestrina) were approved by CSIRO livestock industries Animal Ethics Committees (approval number 1315) and cared for in accordance with Australian National Health and Medical Research Council guidelines. Macaques were sedated with Ketamine prior to any procedures and euthanized with Phenobarbitone prior to any SIV-related disease. Animals were purchased from the NHMRC-supported Australain macaque breeding facility and housed in large cages 3 m×2 m×1 m in groups of 2–4 animals. They were maintained in a 12 hr/12 hr light/dark cycle and given ad libitum access to water. Fresh fruit and other feed including specific monkey chow was provided at least daily along with a dedicated program of food and non-food animal enrichment activities. The animals were monitored daily be experienced animal technicians and remained well throughout the experiments.

### Sorting resting CD4 T cells

In order to analyse SIV DNA turnover we sorted resting CD4+ T cells as previously described [24]. Briefly, frozen PBMC were thawed and stained with live/dead (Near Infra Red–IR (APC-Cy7) viability stain before surface staining with an antibody cocktail of CD69-APC (clone L78), CD3-PE (clone SP34-2), CD4-FITC (clone L200), CD25-APC (clone BC96) and HLA-DR PerCP Cy5.5 (clone L243) on ice. Sorting of fixed resting CD4+ T cells (positive for CD3 and CD4 and negative for HLA-DR and CD69 and CD25) was performed on the FACSAria. Resting CD4+ T cells were HLA-DR- CD69- CD25- CD4+CD3+ T lymphocytes [46]–[49].

### Nested pyrosequencing of SIV DNA in resting CD4+ T cells

DNA from FACS sorted resting CD4+ T cells was extracted using the Qiagen mini DNA kit. Nested KP9-specific pyrosequencing consisted of a first round Gag specific PCR, followed by a series of unique KP9-specific PCR using MID-tagged oligonucleotides (available upon request). Individual combinations of forward and reverse KP9-specific MID-tagged oligonucleotides were employed for each serial animal-time point, as previously described [31].

Nested KVA10-specific pyrosequencing consisted of a first round Tat specific PCR covering the KVA10 SIV Tat_114–123_ epitope (amino acid sequence KKETVEKAVA) using 400 nM of the Tat2 forward primer #541 (5′- CTAGAAGAGGCACAAATTCAACAAGAGAAG-3′) and the Tat2 reverse primer #543 (5′- CACCCATATTGTAGGTAGGTCAGTTCAGTC-3′). Second round KVA10-specific PCRs using MID-tagged oligonucleotides (available upon request) were then employed with unique combinations of forward and reverse KVA10-specific MID-tagged oligonucleotides for each individual serial animal-time point. A second Tat epitope, KSA10 was studied in a similar manner (primers and MID-tagged oligonucleotides available on request). PCR conditions are as described previously [31].

PCR products were visualised on a 1% agarose gel. DNA from PCR products was excised and purified gel extractions were performed using the QIAgen Gel Extraction kit (Qiagen, Valencia, CA). Amplicons were pooled at equimolar ratios and sequenced using the Roche 454 system. The frequency of the WT and EM sequences within the three epitopes (Gag KP9, Tat KVA10 and Tat KSA10) was analysed using a custom software program in BioRuby [50]–[53]. Sequence reads were translated and aligned into six reading frames and compared to a reference SIV sequence using BLAT as previously described [31], [53], [54]. Sequences were first filtered to remove sequences that were not full length or which contained ambiguous nucleotides, and then aligned against the reference sequence to identify WT and EM sequences present in the plasma virus or resting cell DNA.

### Analysis - the model of SIV DNA turnover

The basis for estimation of SIV DNA turnover in resting infected cells is a simple model in which the cells infected by virus (*I_W_* and *I_E_* for the cells infected with WT or EM respectively) become resting (*R_W_* and *R_E_* respectively) at a fixed rate *μ* and disappear from the resting pool at the turnover rate δ:
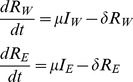
(Eq.1)We assume that plasma virus (*W* for wild type and *E* for escape mutant) closely follows productively infected cells [55], so that *μI_W_* = *fW* and *μI_E_* = *fE*, where *f* is a constant different from *μ*.

Our measured data contain the fraction of WT in resting infected cells and in plasma (we call “escape mutant” all strains at a given epitope that differ from WT), and total viral load, and we want to find the turnover rate *δ* by fitting. Therefore we rewrite Eq.1 in terms of the fraction of WT in resting cells *b_W_* = *R_W_*/(*R_W_*+*R_E_*) and a “dummy variable Λ = (*R_W_*+*R_E_*)/*f*:
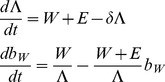
(Eq.2)
Eq.2 has only one fitting parameter, the turnover rate *δ*. We fit this parameter for each animal by choosing *δ* from the interval (0, 2) so that it minimizes the expression
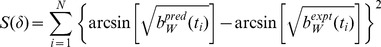
(Eq.3)where *b_W_^pred^* and *b_W_^expt^* are the predicted and the measured values of the fraction of WT in resting infected cells. The fitting method is described in detail in Reece *et al.* [24].

Animals were discarded if there was no data for %WT in the resting cells, if there was little or no escape or if the points were scattered so that the sum of square errors of the fit differed little for all values of the fitting parameter δ in the whole interval (0,2). Consequently, a smaller number of data points was available for fitting the resting CD4+ T cell SIV DNA half-life using pyrosequencing data compared to qRT-PCR measurements.
